# SARS-CoV Infection in a Restaurant from Palm Civet

**DOI:** 10.3201/eid1112.041293

**Published:** 2005-12

**Authors:** Ming Wang, Meiying Yan, Huifang Xu, Weili Liang, Biao Kan, Bojian Zheng, Honglin Chen, Han Zheng, Yanmei Xu, Enmin Zhang, Hongxia Wang, Jingrong Ye, Guichang Li, Machao Li, Zhigang Cui, Yu-Fei Liu, Rong-Tong Guo, Xiao-Ning Liu, Liu-Hua Zhan, Duan-Hua Zhou, Ailan Zhao, Rong Hai, Dongzhen Yu, Yi Guan, Jianguo Xu

**Affiliations:** * Guangzhou Municipal Center for Disease Control and Prevention, Guangdong, People's Republic of China; † National Institute for Communicable Disease Control and Prevention, Beijing, People's Republic of China; ‡ State Key Laboratory of Infectious Diseases Prevention and Control, Bejing, People's Republic of China; § University of Hong Kong, Hong Kong Special Administrative Region, People's Republic of China

**Keywords:** Severe acute respiratory syndrome, coronavirus, palm civet, research

## Abstract

Epidemiologic investigations showed that 2 of 4 patients with severe acute respiratory syndrome (SARS) identified in the winter of 2003–2004 were a waitress at a restaurant in Guangzhou, China, that served palm civets as food and a customer who ate in the restaurant a short distance from animal cages. All 6 palm civets at the restaurant were positive for SARS-associated coronavirus (SARS-CoV). Partial spike (S) gene sequences of SARS-CoV from the 2 patients were identical to 4 of 5 S gene viral sequences from palm civets. Phylogenetic analysis showed that SARS-CoV from palm civets in the restaurant was most closely related to animal isolates. SARS cases at the restaurant were the result of recent interspecies transfer from the putative palm civet reservoir, and not the result of continued circulation of SARS-CoV in the human population.

## Abstract 2

Contact with food animals was associated with SARS-CoV infection in the People’s Republic of China.

The severe acute respiratory syndrome (SARS) epidemic emerged in 2003 in 6 municipalities in the Pearl River delta region in Guangdong, China. Early case-patients were more likely to be persons with occupational exposure to animals, such as animal sellers or restaurant cooks (*1**,**2*). Tracing the source of infection has been complicated, given the sporadic nature of index cases without a clear history of contact with animals. After the World Health Organization (WHO) declared the end of the SARS epidemic, 4 new cases of SARS were reported from December 16, 2003, to January 1, 2004, in Guangzhou in Guangdong Province. These cases were not linked to any laboratory accidents. All patients had a temperature >38°C, radiographic evidence of pneumonia, and serologic evidence of SARS infection. Fever lasted from 6 to 18 days (median 7), no mechanical ventilation was required, and the clinical course of the disease ranged from 21 to 24 days with full recovery. All 4 patients had community-acquired infections without any apparent epidemiologic link. A total of 257 contacts, including 113 close contacts, of these patients were observed for 2 weeks, with no secondary transmission identified. These patients had mild symptoms and no secondary transmission, which was remarkably different from patients in the 2003 epidemic.

Since potential reemergence of SARS leading to epidemic spread was possible, identification of the infectious source was a high priority. The S gene sequence of SARS-associated coronavirus (SARS-CoV) isolated from 2 of these 4 patients was found to be closely related to the sequence of virus isolated from palm civets (*3*). However, 1 of these patients reported no contact with palm civets or other animals in the preceding 2 months. The second patient was a 20-year-old waitress from a restaurant that served palm civets as food (*4**,**5*). Based on the virologic and epidemiologic findings, provincial officials took aggressive action on January 5, 2004, ordering a sweep through farms and food markets to destroy any animals that might harbor SARS-CoV. No additional SARS cases have since been reported. This information highlights the necessity for investigating restaurants as a possible source of infection, understanding that the virus can be transmitted from animals or environmental sources to humans, and clarifying the genetic basis of pathogenicity and infectivity of SARS-CoV from animal sources.

## Methods

### Specimen Collection

Serial nasopharyngeal, fecal, and serum specimens of patients were collected at hospitals by Guangzhou Municipal Centers for Diseases Control and Prevention. When possible SARS was diagnosed in the waitress on January 2, 2004, serum, throat and rectal swabs were obtained from all 6 palm civets at the restaurant. It was reported that the animals were purchased from Xinyuan live animal wholesale market in Guangzhou. Serum samples from employees of the restaurant were obtained on January 4. Persons with positive results provided additional samples as needed. All specimens were stored at –80°C.

### Laboratory Diagnosis and Direct Sequencing of Primary Specimens

Serum samples were tested by enzyme-linked immunosorbent assay (ELISA), immunofluorescent antibody (IFA) test, and Western blot for specific immunoglobulin G (IgG) and IgM. Nasopharyngeal, throat, and rectal specimens were tested by reverse transcription–polymerase chain reaction for polyprotein (P) and nucleocapsid (N) genes of SARS-CoV. Gene sequences were determined directly from original samples. RNA was transcribed into cDNA (SuperScript, Invitrogen, Carlsbad, CA, USA) and subsequently used for PCR amplification. Complete spike (S) gene and whole genome sequencing of SARS-CoV virus was conducted by using 48 primer sets based on the sequence data of a SARS-CoV SZ3 isolate from palm civet (*6*) and an ABI 3730 Genetic Analyzer (Applied Biosystems, Foster City, CA, USA). Assembled genome sequences were compared with those of the first virus isolates of human (TOR2) and animal (SZ3) origin. Any nucleotide (nt) differences were double-checked and confirmed. Sequences from this study were deposited in GenBank (accession nos. AY572034–572038).

### Virus Isolation and Characterization

Samples from patients and animals were cultured in fetal rhesus kidney (FRhK-4) cells or Vero E6 cells for virus isolation as described (*6**,**7*). Cells with or without SARS-CoV virus infection were harvested and fixed in 2.5% glutaraldehyde (Electron Microscopy Sciences, Hatfield, PA, USA) for 4 h and post-fixed in 1% osmium tetroxide for 1 h. Cells were then transferred to a 1.5-mL tube and centrifuged at 1,000 rpm for 10 min. The supernatant was removed and a 2% agarose solution (55°C–60°C) was added to the cell pellet. After the agarose solidified, ≈1-mm cubes containing the cell pellet were cut and dehydrated in graded ethanol. The cubes were then embedded in epoxy resin. Ultrathin sections (70 nm) were prepared and stained with uranyl acetate and lead citrate. Sections were examined with a Philips (*Eindhoven*, the Netherlands) EM208S electron microscope.

### Phylogenetic Construction and Data Analyses

Nucleotide and amino acid sequences were aligned by using MegAlign version 6.0 (DNASTAR, Madison, WI, USA). A neighbor-joining tree with bootstrap values was constructed to estimate phylogenetic relationships among sequences. Nucleotide positions were numbered based on the TOR2 SARS virus isolate (GenBank accession no. NC_004718) (*8*).

## Results

### Epidemiologic Findings of Patients

Epidemiologic investigations showed that 2 of the 4 recent SARS patients were linked with the restaurant serving palm civets as food. One patient was a 20-year-old waitress who became ill on December 26, 2003, with suspected SARS was diagnosed on January 2, 2004, and she was classified as a probable SARS patient by local health authorities on January 8, 2004 (*4**,**5*). She denied eating palm civet or being in close contact with them. However, palm civets were found in her work area and she often passed or stood a short distance from the animal cages. The other patient was a 40-year-old physician who ate at the restaurant on December 31, 2003, and first showed symptoms on January 7, 2004. His dining table was within 5 m of civet cages.

Serial serum samples from both patients were positive for IgG and IgM against SARS-CoV by ELISA, IFA, and Western blots. Positions 22907–23192 (286 bp) of the S gene were sequenced from a nasopharyngeal swab isolate from the waitress and from a fecal specimen from the physician (samples were obtained on January 5 and January 12, 2004, respectively). The 2 S gene sequence fragments were identical, but differed from all S gene sequences available on public databases. Attempts to isolate virus from these specimens by using Vero E6 cells were unsuccessful. Isolation of virus with the FRhK-4 cell line was not attempted because the volume of specimen from patients was limited.

### Epidemiologic and Etiologic Findings of the Restaurant

The restaurant is in a 2-story building in downtown Guangzhou. Eight animal cages containing 6 palm civets (*Paguma larvata*) were stacked (2 cages per stack) at the front door of the restaurant. The cages were approximately 1 m from the sidewalk and 2 m from the first row of dining tables on the ground floor of the restaurant. Pedestrians walking in the street and customers dining on the ground floor could easily see the animals in the cages.

Both P and N genes of SARS-CoV were found by nested PCR in all throat and rectal swab specimens from 6 palm civets (Table 1) (*9*). Three complete genome sequences and 2 complete S gene sequences of SARS-CoV were found in rectal or throat swab specimens from 5 of the 6 palm civets (Table 1) (*10*). The 286-bp S gene sequences from isolates from the waitress and the physician were identical to 4 of 5 S gene sequences from palm civets from the restaurant, but differed from other sequences available from public databases (Table 2). SARS-CoV virus was isolated from FRhK-4 cells cultured with a rectal swab specimen of a palm civet, but not from Vero E6 cells. Cytopathic effects (CPE) of SARS-CoV virus on FRhK-4 cells were visible 4 days after culture with a fecal swab sample. Electron microscopy showed typical morphologic features of SARS-CoV virus in a thin section of the infected cell. A complete genome sequence of the SARS-CoV isolated from a palm civet was determined directly from the original sample and submitted to GenBank (accession no. AY572034).

**Table 1:** Detection of severe acute respiratory syndrome–associated coronavirus genes in palm civets*

Palm civet	Nucleocapsid and polyprotein genes		Sequences detected (GenBank accession nos.)
	Throat swab specimen	Rectal swab specimen	
007	+	+	Complete genome (AY572034)
010	+	+	Complete genome (AY572035)
014	+	+	Spike gene (AY572036)
018	+	+	ND
019	+	+	Spike gene (AY572037)
020	+	+	Complete genome (AY572038)

*Nucleocapsid and polyprotein genes were detected by nested reverse transcription–polymerase chain reaction. Spike gene sequences were determined from rectal swab isolates.+, positive; ND, not detected.

**Table 2:** Comparison of signature nucleotide variations in the spike (S) gene of SARS-CoV from various sources*

Virus	Source	Signature nucleotide variation position of S gene†																				
		2	2	2	2	2	2	2	2	2	2	2	2	2	2	2	2	2	2	2	2	2
		1	2	2	2	2	2	2	2	2	2	2	3	3	3	3	3	3	3	4	4	5
		9	5	5	5	8	8	9	9	9	9	9	3	3	3	4	7	7	8	5	9	0
		0	1	2	7	7	7	0	2	2	3	5	1	1	3	8	1	8	2	6	7	3
		7	7	2	0	4	5	6	7	8	0	1	6	7	0	5	9	5	3	6	8	1
Civet007	Civet, restaurant	T	G	G	C	T	T	C	G	A	G	G	T	T	A	C	G	T	G	C	G	C
Civet010	Civet, restaurant	T	G	G	C	T	T	C	G	A	G	G	T	T	A	C	G	T	G	C	G	C
Civet019	Civet, restaurant	T	G	G	C	T	T	C	G	A	G	G	T	T	A	C	G	T	G	C	G	C
Civet020	Civet, restaurant	T	G	G	C	T	C	C	G	A	G	G	T	T	A	C	G	T	G	C	G	C
Civet014	Civet, restaurant	T	G	G	C	T	C	C	A	A	G	G	T	T	A	C	G	T	G	C	G	C
Waitress	Patient 2, waitress								G	A	G	G										
Customer	Patient 4, customer								G	A	G	G										
GD03T0013	Patient 1	T	G	G	C	T	C	C	A	T	G	G	T	T	A	C	G	T	G	C	G	C
SZ3	Civet, market	C	G	G	C	C	C	T	A	A	A	G	G	C	T	C	C	C	G	C	G	T
SZ16	Civet, market	C	G	G	C	C	C	T	A	A	A	G	G	C	T	C	C	C	G	C	G	T
GZ60	Early phase	C	G	G	T	C	C	T	A	T	A	C	G	C	T	C	C	C	G	T	A	T
HGZ8L1-A	Early phase	C	G	G	T	C	C	T	A	T	A	C	G	C	T	T	C	C	G	T	A	T
ZS-A	Early phase	C	G	G	T	C	C	T	A	T	A	C	G	C	T	T	C	C	G	T	A	T
ZS-B	Early phase	C	G	G	T	C	C	T	A	T	A	C	G	C	T	T	C	C	G	T	A	T
ZS-C	Early phase	C	G	G	T	C	C	T	A	T	A	C	G	C	T	T	C	C	G	T	A	T
GD01	Early phase	C	G	G	T	C	C	T	A	T	A	C	G	C	T	T	C	C	G	C	A	T
GZ02	Early phase	C	G	G	T	C	C	T	A	T	A	C	G	C	T	T	C	C	G	T	G	T
HSZ-Bb	Early phase	C	A	A	T	C	C	T	A	T	A	C	G	C	T	T	C	C	G	T	G	T
HSZ-Bc	Early phase	C	A	A	T	C	C	T	A	T	A	C	G	C	T	T	C	C	G	T	G	T
HSZ-Cb	Early phase	C	A	A	T	C	C	T	A	T	A	C	G	C	T	T	C	C	G	T	G	T
HSZ-Cc	Early phase	C	A	A	T	C	C	T	A	T	A	C	G	C	T	T	C	C	G	T	G	T
GZ50	Middle phase	C	A	A	T	C	C	T	A	T	A	C	G	C	T	T	C	C	T	T	A	T
BJ01	Late phase	C	A	A	T	C	C	T	A	T	A	C	G	C	T	T	C	C	T	T	A	T
BJ03	Late phase	C	A	A	T	C	C	T	A	T	A	C	G	C	T	T	C	C	T	T	A	T
HKU-36871	Late phase	C	A	A	T	C	C	T	A	T	A	C	G	C	T	T	C	C	T	T	A	T
HKU-39849	Late phase	C	A	A	T	C	C	T	A	T	A	C	G	C	T	T	C	C	T	T	A	T
HKU-65806	Late phase	C	A	A	T	C	C	T	A	T	A	C	G	C	T	T	C	C	T	T	A	T
CUHK-W1	Late phase	C	A	A	T	C	C	T	A	T	A	C	G	C	T	T	C	C	T	T	A	T
CUHK-Su10	Late phase	C	A	A	T	C	C	T	A	T	A	C	G	C	T	T	C	C	T	T	A	T
Fra	Late phase	C	A	A	T	C	C	T	A	T	A	C	G	C	T	T	C	C	T	T	A	T
Tor2	Late phase	C	A	A	T	C	C	T	A	T	A	C	G	C	T	T	C	C	T	T	A	T
Urbani	Late phase	C	A	A	T	C	C	T	A	T	A	C	G	C	T	T	C	C	T	T	A	T

*Blank spaces indiate information not available. Early, middle, and late phases indicate when virus was isolated from patients in various stages of the 2003 epidemic. SARS-CoV, severe acute respiratory syndrome.

†Numbers indicate position of signature nucleotide variations on the virus genome based on SARS-CoV Tor2 numbering. The 4 signature nucleotide variations in 286-bp S gene sequences for isolates from 2 restaurant-related patients were identical to 4 of 5 S gene sequences of palm civets at the restaurant.

IgG antibodies against SARS-CoV were detected in 2 (5.1%) of 39 employees of the restaurant. This was higher than that observed in the control groups (1%–3%) (*11*). One employee tested positive for IgM against SARS-CoV in serum samples obtained on January 4 and January 13. Results became negative by January 17, 2004, with no illness or fever in the previous 2 months. This employee worked as a head waitress and often helped customers select palm civets from animal cages. A cook in the restaurant also tested positive for IgG antibody to SARS-CoV.

### Nucleotide and Amino Acid Sequence Variations

Comparison of 5 complete S gene sequences (3,768 nt) from palm civets at the restaurant, 22 S gene sequences from SARS patients in the early 2003 epidemic, and 2 viruses isolated from palm civets in 2003 showed 60 nt polymorphisms. Only 5 signature nt variations (SNVs) were observed in the 5 complete S gene sequences from palm civets determined in this study, indicating that SARS-CoV sequences from civets at the restaurant were not different from those of the original animal SARS source. We also observed that 21 SNVs could be used to distinguish viruses with high pathogenicity and infectivity from those with low pathogenicity and infectivity relative to clinical presentation and transmission events (Table 2).

Three of 5 complete S gene sequences from palm civets at the restaurant did not contain any of the 21 SNVs. The remaining 2 isolates (Civet014 and Civet020) had only 1 or 2 SNVs. In contrast, 11 of 22 SARS-CoV strains isolated from humans in Canada, Germany, and Vietnam had all 21 SNV mutations (Table 2). It should be noted that the first human SARS-CoV isolated, GD01, had 17 of 21 SNVs (Table 2). This virus caused severe infections in humans, but did not spread from Guangdong Province (*12*). Similar SNV patterns were observed in other isolates from patients at the beginning of the 2003 epidemic (*13*). Four isolates (ZS-A, SZ-B, SZ-C, and HGZ8L1-A) had 18 of 21 SNVs and were obtained from patients with contact histories traceable to some of the earliest independent cases, but with no further transmission recorded (*13*). Virus GZ02 had 17 SNVs. Another group of 2 early isolates, HSZ-B and HSZ-C, had 19 SNVs, in addition to an 82-nt deletion (*13*). Virus GZ60, which was isolated from nasopharyngeal aspirates of a healthcare worker at Guangdong Chest Hospital on February 18, 2003, had 18 SNVs (*10*). Guan et al. named this virus SARS-CoV subcluster A1, together with GD01 and GZ43 (*10*). None of the sequences of these early-phase isolates have been observed in the middle or later phase of the epidemic, suggesting these isolates had low or mild infectivity (*13*).

When deduced amino acid sequences were analyzed, 15 signature amino acid variations (SAAVs) were observed that could distinguish between viruses with low or high pathogenicity and infectivity. Three of the 5 recent SARS-CoV isolates from palm civets had no SAAVs, while viruses isolated from outbreaks in various countries had all 15 SAAVs (*7**,**10**,**14**,**15*). The isolates from the early phase of the 2003 epidemic (GD01, ZS-A, SZ-B, SZ-C, HGZ8L1-A, HSZ-B, HSZ-C, and GZ60) had 12 or 13 SAAVs (*6**,**7**,**13*). SARS-CoV SZ3 and SZ16 had 7 SAAVs mutations (*6*). The S protein sequence predicted for the first SARS case of 2003–2004 had only 3 SAAVs (*13*).

### Genomic Differences

When the complete genome sequences of SARS-CoV determined in specimens from palm civets at the restaurant (n = 3), animal markets (n = 2), and patients (n = 23) were compared, the 29-nt deletion (positions 27869–27897) was absent in all isolates from palm civets at the restaurant and at the market, but was present in 22 of 23 patient isolates. The only human isolate (GZ01) without the 29-nt deletion was from a patient in the 2003 epidemic. In addition to S gene sequences, another 42 SNVs were identified, of which 33 were located on the gene encoding P protein (open reading frame [ORF] ab), and on 9 other genes for uncharacterized proteins: ORF 3 (5 SNVs), membrane protein (2 SNVs), and N protein (2 SNVs). However, when complete genome sequences of SARS-CoV from palm civets at the restaurant were compared with those of isolates from palm civets from the market, only 37 SNVs were identified and located on genes encoding P protein (20 SNVs), S protein (11 SNVs), ORF 3a (3 SNVs), M protein (1 SNV), and N protein (2 SNVs). All nucleotide changes were observed in virus sequences of palm civets from the market, but not in virus sequences of animal isolates from the restaurant.

### Phylogenetic Analysis

Analysis of the S gene of SARS-CoV showed that viral isolates of animal origin clustered into 2 distinct groups. Group A is represented by SZ3 and SZ16, which were isolated from palm civets in 2003. Group B is represented by viruses found in palm civets at the restaurant (Figure) (*6*). Analysis of complete genome sequences showed the same relationships (data not shown). These most recent SARS patients were therefore infected by SARS-CoV that is most closely related to virus isolates from palm civets at the restaurant (Figure) (*6*).

**Figure:**
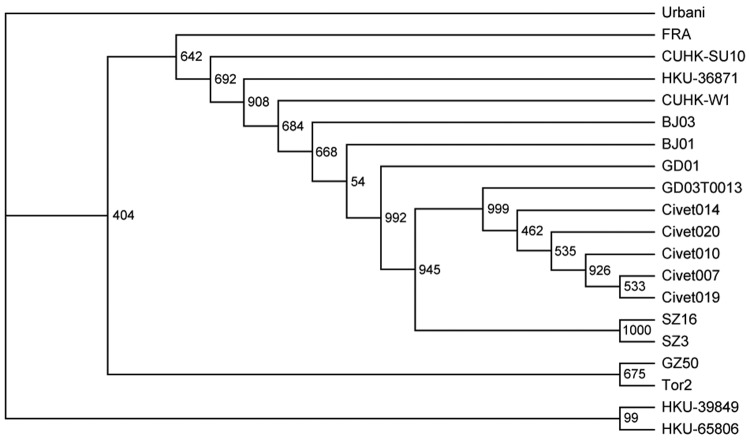
Phylogenetic relationships of severe acute respiratory syndrome (SARS) virus isolates based on the spike gene. The neighbor-joining tree was constructed by the neighbor-joining process with 1,000 bootstrap replicates. The origins of the sequences are as follows: Civet007, Civet010, Civet019, Civet020, and Civet014, palm civets from the restaurant; GD03T0013, the first SARS patient in 2004; SZ3 and SZ16, palm civets from a Shenzhen market in 2003; GZ60, HGZ8L1-A, ZS-A, ZS-B, ZS-C, and GD01, early phase isolates in 2003 without the 29-nucleotide (nt) deletion; GZ02, HSZ-Bb, HSZ-Bc, HSZ-Cb, and HSZ-Cc, early phase isolates from the 2003 epidemic with an 82-nt deletion; GZ50, HKU-36871, HKU-39849, HKU-65806, CUHK-W1, CUHK-Su10, BJ01, BJ03, Fra, Tor2, and Urbani, middle and late phase isolates from the 2003 epidemic.

## Discussion

The source of SARS-CoV, how it was introduced into humans, and where it may reemerge are critical questions related to disease control (*16*). Understanding the mode of transmission of SARS-CoV into humans is essential in designing appropriate prevention and control measures for future SARS epidemics. We provide the first direct evidence that SARS-CoV was transmitted from palm civets to humans, and that a restaurant serving palm civets positive for this virus was the source of infection for 2 of 4 confirmed SARS patients during the resurgence of SARS in the winter of 2003–2004 (*6**,**17*).

All 6 palm civets from the restaurant were positive for SARS-CoV. Partial S gene sequences were identical in both patients from this study and to 4 of 5 S gene sequences from palm civets from the restaurant, but different from more than 100 S gene sequences from SARS patients worldwide (*6**,**10**,**12**,**13*). That the restaurant was an infection source was further supported by serologic investigation of restaurant employees. Specific IgG was detected in 2 of 39 employees, 1 with a history of close contact with these palm civets. However, we lack evidence that eating civet could transmit the virus because the employees had not eaten palm civet before SARS developed. The patients most likely were infected by close exposure to animals carrying SARS-CoV in the restaurant. This situation may be similar to those earliest index cases linked to markets or restaurants that occurred in winter of 2002–2003 (*18*). Results of PCR tests conducted by the WHO were positive for SARS-CoV in specimens from the bottom of animal cages and the kitchen of the restaurant (*19*).

Genome sequence analysis data strongly suggest that sporadic cases of SARS in Guangzhou in 2003–2004 were caused by SARS-CoV of animal origin. The 29-nt deletion was not observed in palm civets from the restaurant, but was present in almost all human isolates, and may have resulted from the adaptation and evolution of SARS-CoV in humans. SNVs in S gene sequences have been reported in several studies of the molecular evolution of SARS-CoV (*6**,**7**,**12**,**13*). The characteristic SNV pattern of S genes has 21 nt. SARS-CoV isolated from palm civets at the restaurant had 0, 1, or 2 SNVs. However, viruses from several provinces of China and other countries had all 21 SNVs (*7**,**10**,**14**,**15*). Viruses isolated in the early phase of the 2003 epidemic had 16–19 SNVs (*6**,**7**,**13*). The SZ3 and SZ16 isolates from palm civets in 2003 had 11 SNVs (*6*), while the S gene from the first case of SARS encountered in 2004 had only 3 SNVs (Table 2) (*13*). When the complete genomes of SARS-CoV from palm civets at the restaurant were compared with sequences of human isolates, 62 SNVs were identified. However, when the complete genome was compared with sequences of virus isolated from palm civets from animal markets in the 2003 epidemic, only 37 SNVs were identified.

Phylogenetic analysis of the S gene of SARS-CoV also showed that viruses from palm civets at the restaurant were more closely related to previously described viruses of animal origin, and these were more closely related to viruses isolated from patients during the early epidemic phase. Moreover, all SARS-CoV strains, including isolates from animal markets, had evolved from isolates in palm civets at the restaurant (Figure). Clearly, SARS cases contracted at the restaurant were the result of recent interspecies transfer from a putative palm civet virus reservoir, rather than the result of circulation of SARS-CoV in the human population.

SNV and phylogenetic analysis also suggest that the virus responsible for SARS infections in 2004 was not yet able to cause severe disease in humans. Minor clinical symptoms and no subsequent transmission have been recognized as features of the recent SARS infections. These findings support our observations that SARS-like illness did not develop in any of the 257 contacts of the 4 patients, or in any of the health care workers attending them. However, epidemiologic data can only provide clues to the biologic characteristics of the virus. Therefore, experimental infection using animal models is necessary to measure the relative pathogenic potential of various strains of SARS-CoV isolated from human and animals.
